# Design and evaluation of a voltage-controlled current source for galvanic vestibular stimulation research

**DOI:** 10.1016/j.ohx.2025.e00647

**Published:** 2025-04-10

**Authors:** Zhi Liu, Shieru Suzuki, Tatsuki Fushimi, Yoichi Ochiai

**Affiliations:** a Graduate School of Comprehensive Human Sciences, University of Tsukuba, Tsukuba, 305-8550, Japan; b Faculty of Library, Information and Media Studies, University of Tsukuba, Tsukuba, 305-8550, Japan; c R&D Center for Digital Nature, University of Tsukuba, Tsukuba, 305-8550, Japan; d Pixie Dust Technologies, Inc, Tokyo, 101-0061, Japan

**Keywords:** Galvanic vestibular stimulation, Voltage-controlled current source, Mix-reality application, Psychophysics

## Abstract

Galvanic Vestibular Stimulation (GVS) is a non-invasive technique that stimulates the vestibular system, which is crucial for maintaining balance and processing spatial orientation. The integration between the visual and vestibular systems, known as Oculo-Vestibular Recoupling (OVR), has shown promising potential in reducing motion sickness and enhancing immersion in Extended Reality (XR). However, a noticeable challenge in GVS research is the lack of open-sourced devices, with most studies relying on self-made prototypes or constant current power supplies. The prototyping of such devices necessitates rigorous testing and calibration, processes that are both resource-intensive and time-consuming. These challenges are particularly pronounced for researchers with limited expertise in electronics, thereby increasing the safety risks and complicating the reproducibility of experimental results. To address these issues, this paper introduces an open-source voltage-controlled current source (VCCS) module specifically designed for GVS. The proposed module provides a safe, stable, and compact solution. This study details the hardware development, performance evaluation, and wireless integration of the module, as well as a simple control methodology. Furthermore, a small-scale user study is conducted to validate the feasibility and user perception of GVS using the proposed module. This comprehensive approach aims to offer an easily accessible solution for researchers engaged in GVS-related studies.

## Graphical abstract

**Figure:**
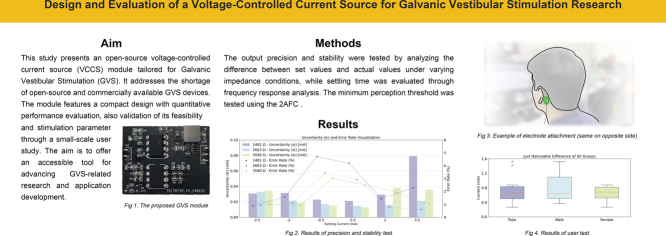

## Specifications table

*Please replace the italicizied instructions in the right column of the table with the relevant information about your hardware.*

**Table:** 

**Hardware name**	*Voltage controlled Howland current source for galvanic vestibular stimulation*

**Subject area**	• *Engineering and material science* • *Medical (e.g. pharmaceutical science)* • *Neuroscience* • *Educational tools and open source alternatives to existing infrastructure*

**Hardware type**	• *Electrical engineering and computer science* • *Mechanical engineering and materials science*

**Closest commercial analog**	• *Soterix Medical, GVS headset, see: “**https://soterixmedical.com/research/vestibular**”* • *BrainPatch, GVS headphone, see: “**https://brainpatch.ai**”*

**Open source license**	*CERN Open Hardware Licence Version 2 - Weakly Reciprocal*

**Cost of hardware**	*Around $22 USD.*

**Source file repository**	*The hardware specifications, software, and datasets generated and analyzed during this study are openly available at Zenodo:*https://doi.org/10.5281/zenodo.14545604.

**OSHWA certification UID**	*JP000020*

## 1 Hardware in context

Galvanic Vestibular Stimulation (GVS) is a non-invasive method used to stimulate the vestibular system [1]. It is a variant of transcranial direct current stimulation (tDCS), in which electrodes are attached to mastoids behind the ears, and an electrical stimulus is applied by a low-amperage pulsed direct current, to stimulate and inhibit the vestibular system [2], [3], [4], [5]. Change in vestibular input can create a sensation of virtual head motion and affect balance to evoke body tilt [6], [7], modulates posture and balance [8], [9]. For example, research has shown that GVS significantly reduces postural sway induced by mechanical perturbations, allowing users to maintain a more stable and upright stance [10]. Furthermore, in relation to noisy galvanic vestibular stimulation (nGVS), a study by Matsugi et al. investigated its effects on postural control, reporting significant increases in body sway and soleus muscle activity, particularly under unstable conditions and independently of visual input [11]. It has been used as a rehabilitation method in medical fields such as improving body balance [12], sea sickness [13], bilateral vestibulopathy [14] and traumatic spinal cord injury [15]. The artificial tilt sensation also shows great potential to replace expensive and complex motion platforms used in aviation [16], [17], [18] and sports training [19], [20]. For instance, a study conducted by Scinicariello et al. demonstrated that pseudorandom bilateral bipolar GVS effectively replicates microgravity-induced decreases in the landing performance of astronaut pilots, significantly increasing touchdown speeds and landing errors during Shuttle simulations [21].

More recently, GVS has also gained attention not only in medical and training fields but also in the entertainment industry. In the Extended Reality (XR) industry, one of the significant challenges is the over-emphasis on visual feedback in current virtual environments. This limitation often results in an incomplete sensory experience, which can lead to a mismatch between the visual stimuli that users perceive and the physical sensations their bodies register, as explained by the sensory conflict theory [22]. Such discrepancies can detract from the sense of presence and immersion that virtual reality aims to deliver, while also contributing to discomforts such as dizziness and nausea, commonly referred to as cybersickness. Recent research integrating GVS with XR has shown encouraging results in addressing these challenges. Studies have demonstrated that GVS can effectively mitigate cybersickness symptoms [23], enable techniques such as redirected walking for enhanced spatial exploration [24], [25] and improve overall immersion across diverse virtual scenarios [26]. This multidisciplinary approach highlights the potential of combining multiple sensory inputs to create more immersive and comfortable XR experiences.

Currently, studies related to GVS often rely on self-made prototypes and stationary power sources, with a noticeable issue being the absence of standardized, compact, and easily accessible open-source modules. Prototyping in such devices requires the integration of hardware and software design, along with testing and calibration processes, making it both resource intensive and time consuming. Inefficiencies or deficiencies in the apparatus can lead to inconsistencies in operational parameters, which pose not only safety risks, but also compromise the validity of experimental outcomes.

Based on the analysis of studies combining GVS and XR, we prioritize precision, customizability, and portability as key objectives in development. The precision of the stimuli delivered (current intensity) directly impacts both the efficacy and safety of GVS [27], therefore maintaining precise control is critical. Safety is established for low-intensity transcranial electrical stimulation (tES) defined as less than 4 mA[28]. However, in practice, tissue impedance is a dynamic variable influenced by multiple factors, including stimulation frequency [29], blood circulation, and skin hydration [30]. Evidence also indicates that impedance decreases during the initiation of tDCS [31]. To achieve precise current stimulation over tissues, current sources are generally considered to be a preferable alternative to voltage sources, as they provide constant stimulation parameters regardless of variations in tissue impedance. This ensures consistent outcomes while reducing the risk of over-stimulation. On the other hand, the ability to fine-tune stimulation parameters, such as current amplitude, frequency, and waveform shape, is also important for advancing research and applications. This flexibility allows researchers to tailor stimulation to specific experimental requirements or design objectives, enabling the exploration of new stimulation protocols that may lead to new insights [32]. Beyond hardware performance, size and weight are also practical considerations in the design of this module. A lightweight and compact module can be easily integrated with other devices, enhancing its versatility. Furthermore, for experiments conducted outside laboratory settings, particularly those that require participants to move freely or maintain natural postures, portability is essential to ensure usability and minimize interference with experimental conditions.

To meet these requirements, we designed a compact and portable current source capable of generating precisely controlled arbitrary waveforms through software-defined signal patterns. The design is based on the Howland Current Source (HCS), chosen for its ability to deliver accurate and symmetric bidirectional currents [33]. To provide an intuitive and efficient control method, the module incorporates a voltage-controlled interface, where an input voltage is linearly mapped to an output current range of ±4 mA, allowing users to define and implement complex stimulation patterns with high accuracy. In addition, this approach minimizes the need for discrete components, ensuring a compact and scalable design suitable for various applications.

The module was evaluated through both hardware performance tests and user studies, demonstrating its effectiveness and reliability. The results confirmed consistent delivery of the desired current levels with minimal variability under varying impedance loads. Furthermore, user testing established the perceptual threshold for tilting perception, providing a reference for future experimental settings. Our study contributes by introducing an open-source, low-cost, customizable, and reproducible Galvanic Vestibular Stimulation (GVS) device, while also providing comprehensive evaluation data that serve as a reference for future research and device development. The existing literature often lacks the technical details necessary for reproducibility and omits rigorous performance assessments; our work ensures transparency and accessibility by openly sharing the evaluation setup and source code. By lowering technical barriers, this study aims to facilitate more efficient prototyping and safer experimentation, ultimately enabling the broader adoption and advancement of GVS technology.

**Fig. 1:**
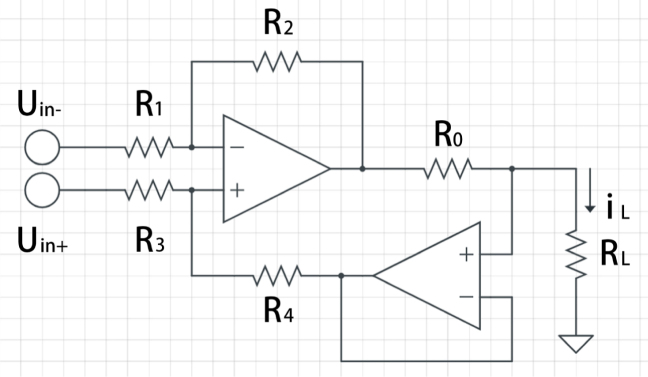
Using MT3608 to turn 3.7 V into 24.6 V.

## 2 Hardware description

The HCS was first invented by Prof. Bradford Howland in 1962 (published in 1964), and it is an operational amplifier-based circuit configuration that converts the linear differential voltage into a corresponding current [34]. Previous research has demonstrated its application in making a low-consumption wireless neuro stimulator [35].

**Fig. 2:**
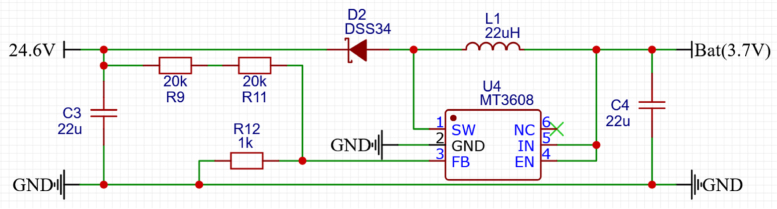
Enhanced Howland Current Source (EHCS).

However, conventional HCS has several weaknesses: the output node cannot swing close to the supply rails, reducing its output range; it exhibits nonlinear offset voltage, especially near rail-to-rail inputs; driving high load voltages results in significant power wastage, particularly for large swings; and unequal resistor values for heavy loads can cause errors due to input bias current in bipolar operational amplifiers (op-amps) [36]. Subsequent studies introduced modifications to improve DC precision and drive capability. Jiang [37] proposes a composite amplifier design, shown in Fig. 2, which incorporates an additional buffer stage to ensure fast settling speed and high accuracy. Our study references this design as the foundational basis for the GVS module.

The design of a HCS requires a predefined target impedance value. Few previous studies have provided a reference value for human impedance between mastoids [38], it is significantly varied due to individual differences in tissue impedance and other influencing factors. To accommodate this variability, we set a wide impedance range from 0 Ω to 5 kΩ to ensure the versatility of the module between different users. Regarding safety limitation, we refer to previous studies on direct current transcranial stimulation, the safety thresholds guideline by Antal et al. which limit output within ±4 mA[28].

The voltage requirement can be calculated by:

(1) $$V_{\text{required}}=I_{\text{max}}\cdot R_{\text{max}}+V_{\text{headroom}}$$

The GVS module itself requires minimal:

(2) $$V_{\text{required}}=I_{\text{max}}\times R_{\text{max}}=\left(4\times 10^{-3}\right)\times \left(5000\right)=20\;V$$

Sense resistor from INA219 requires:

(3) $$V_{\text{required}}=I_{\text{max}}\times R_{\text{sense}}=\left(4\times 10^{-3}\right)\times \left(100\right)=0.4\;V$$

$V_{\text{requirement}}$ is the required voltage (V), $I_{\text{max}}$ is the maximum current (A) limited within ±4mA, $R_{\text{max}}$ is the maximum load impedance (Ω) ranging from 0Ω to 5kΩ, $R_{\text{sense}}$ is the resistance of the sensing resistor (Ω) and $V_{\text{headroom}}$ is the additional voltage margin (V) necessary for stable operation. According to the LT1013 datasheet [39], the maximum peak output voltage swing is 3 V lower than the power supply (evaluated over the full temperature range, with a supply voltage of ±15 V under a load of 2 kΩ). Based on Eqs. (2), (3), our module requires a minimum operating voltage of 20.4 V. Therefore, $V_{\text{headroom}}$ is expected to exceed 3 V. In our prototype, to ensure that the supply voltage can accommodate all potential variations, including component tolerances and additional circuit losses, we set the supply voltage as 24.6 V.

To improve portability and accessibility, this module is designed to operate directly from a standard 3.7 V battery source. To achieve this, a DC-DC booster (MT3608) is used to increase the supply voltage to 24.6 V, ensuring that the module avoids clipping or distortion at maximum output (shown in Fig. 1). This integration also prevents possible power mismatches, which could result in insufficient current output at higher impedance or exceeding the op-amp’s voltage limits. The output voltage of the MT3608 can be calculated using the equation:

(4) $$V_{\text{out}}=0.6\left(1+\frac{R_{9}+R_{11}}{R_{12}}\right)$$

**Fig. 3:**
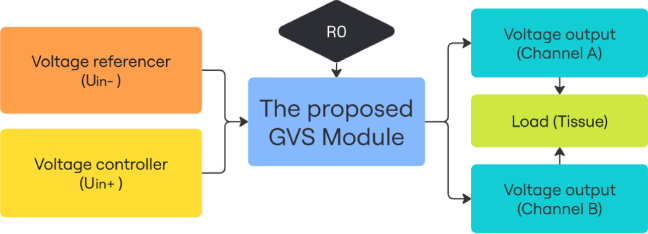
Schematics of the proposed module.

**Fig. 4:**
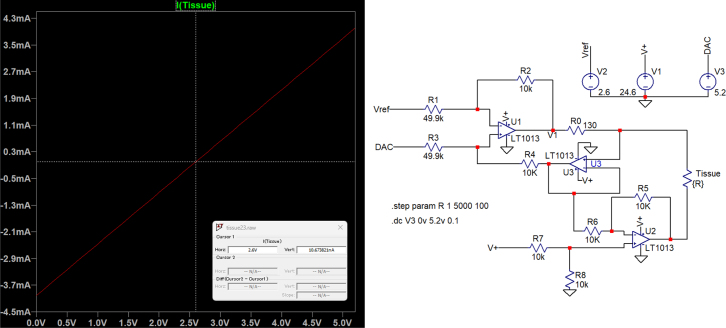
Simulated output (left) and circuit diagram of the GVS module (right).

Based on the previous work of EHCS [37], if the resistor ratios are defined as:

(5) $$\frac{R_{1}}{R_{2}}=\frac{R_{3}}{R_{4}}=k$$

The current output of $i_{\text{L}}$ (A) can be calculated using:

(6) $$i_{L}=\frac{u_{in+}-u_{in-}}{kR_{0}}$$

As specified in Fig. 3. In this design, op-amps (LT1013) are powered by the integrated boost converter (Fig. 1). While using a n-bit Digital-to-Analog Converter (DAC) as $U_{\text{in+}}$ and a voltage referencer half the maximum DAC output as $U_{\text{in-}}$, assuming the maximum intended output current on a single side is N mA, the relationship of the given voltage $V_{\text{DAC}}$ and the output current $I_{\text{out}}$ (mA) can be described by:

(7) $$I_{\text{out}}=N\frac{V_{\text{DAC}}}{V_{\text{ref}}}-N$$

Current output($I_{\text{out}}$) can be adjusted dynamically by changing the voltage at the non-inverting input ($V_{\text{DAC}}$). Users are free to choose their own way to configure $V_{\text{DAC}}$, depending on the desired granularity and available hardware. The reference voltage ($V_{\text{ref}}$) at the inverter input should be half of the maximum ($V_{\text{ref}}$) to ensure symmetric control over the current output ($I_{\text{out}}$).

In this way, by adjusting the DAC’s input code ($D_{n}$) the output current can be controlled. The relation between $D_{n}$ and the output current $I_{\text{out}}$ (mA) can be expressed as:

(8) $$I_{\text{out}}=\frac{8}{2^{n}-1}D_{n}-4$$

Using the circuit diagram proposed in Fig. 4, this module is capable of producing a linear and stable output current ranging from −4mA to 4mA. To allow for precise measurement of parameters such as voltage and current at each step during the design and testing process, a 5.2 V power supply (Adafruit PowerBoost 500) and a 12-bit DAC module (Adafruit MCP4725) were used to regulate the input voltage of the DAC, allowing the output current to be divided into 4096 steps.

**Fig. 5:**
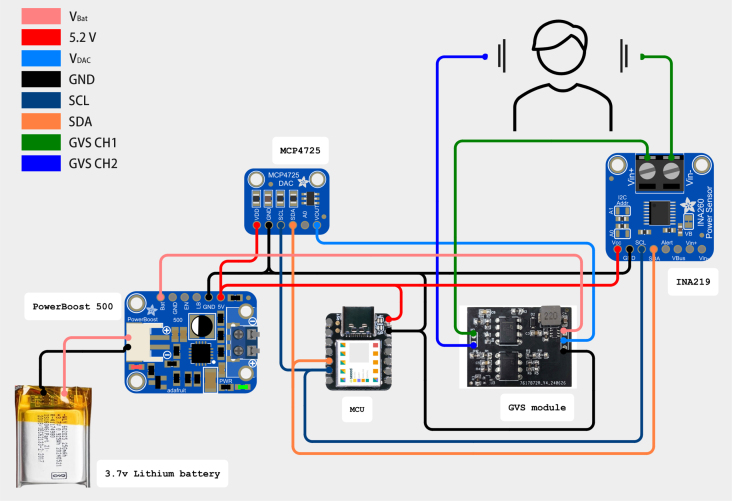
Circuit diagram for module evaluation.

In addition, a current sensor (Adafruit INA219) was incorporated in our test to provide real-time monitoring of the current intensity along with the corresponding variation curve. The real-time current sensor also allows the implementation of a programmable safety protocol, enabling users to define a maximum allowable current threshold based on their specific needs, avoiding the current to exceed the user defined threshold. The reference voltage of 2.65 V is set as half of the maximum DAC output voltage to achieve symmetric current control. The illustration of the setup is shown in Fig. 5.

These settings are not mandatory. As mentioned above, the module’s advantage lies in its ability to provide linear control of output current directly via voltage. This supports DAC-based control and allows integration with devices such as strain gauges, thermistors, and pressure sensors, enabling current modulation for applications such as real-time interactions, adaptive feedback, and sensor-driven automation.

This module addresses the growing interest in the application of GVS technology beyond medical uses, particularly in the field of human–computer interaction (HCI), where stability, customizability, and mobility are crucial for developing interactions. Currently, few commercial products address these needs, researchers are often spending significant time prototyping rather than focusing on their core research, diverting their attention and resources away from the primary objectives. This study provides a compact and stable GVS module with a user-friendly control interface. Making GVS more accessible for HCI related research.

## 3 Design files summary

**Table:** 

Design filename	File type	Open source license	Location of the file
*Schematic diagram*	*ASC file*	*CERN-OHL-W-2.0*	https://doi.org/10.5281/zenodo.14545604
*Bill of materials (BOM)*	*CSV file*	*CERN-OHL-W-2.0*	https://doi.org/10.5281/zenodo.14545604
*Arduino script (demo 1 to 3)*	*INO file*	*MIT*	https://doi.org/10.5281/zenodo.14545604
*Interface (Unity)*	*Bundle*	*MIT*	https://doi.org/10.5281/zenodo.14545604
*Circuit diagram*	*PNG file*	*CERN-OHL-W-2.0*	https://doi.org/10.5281/zenodo.14545604
*Gerber file*	*ZIP file*	*CERN-OHL-W-2.0*	https://doi.org/10.5281/zenodo.14545604
*Testing program (Python)*	*Python*	*MIT*	https://doi.org/10.5281/zenodo.14545604
*Experiment data*	*CSV file*	*MIT*	https://doi.org/10.5281/zenodo.14545604
*LTSpice simulation*	*ASC file*	*MIT*	https://doi.org/10.5281/zenodo.14545604

## 4 Bill of materials summary

**Table:** 

Designator	Component	Number	Cost per unit (USD)	Total cost (USD)	Source of materials	Material type
C3, C4	22 µFCapacitor	2	0.02	0.04	LCSC	Electronic
C5, C6	200 pFCapacitor	2	0.22	0.44	LCSC	Electronic
D2	DSS34 Diode	1	0.07	0.07	LCSC	Electronic
H2	Header 2.54 mm 1x2	1	0.04	0.04	LCSC	Electronic
J1	Header 2.54 mm 1x3	1	0.14	0.14	LCSC	Electronic
L1	22 µHInductor	1	0.14	0.14	LCSC	Electronic
R1	100 Ω	1	0.001	0.002	LCSC	Electronic
R1P	30 Ω	1	0.001	0.001	LCSC	Electronic
R12	1 kΩ	1	0.001	0.001	LCSC	Electronic
R2, R4, R6, R7, R8, R14	10 kΩ	6	0.05	0.1	LCSC	Electronic
R3, R5	49.9 kΩ	2	0.06	0.12	LCSC	Electronic
R9, R11	20 kΩ	2	0.01	0.02	LCSC	Electronic
U3	ISL60002 Voltage Ref.	1	4.34	4.34	DigiKey	Electronic
U4	MT3608 DC-DC Booster	1	0.06	0.06	LCSC	Electronic
U1, U2	LT1013CN Op-Amp	2	7.77	15.54	LCSC	Electronic
PCB	PCB manufacture	1	0.5	0.5	JLCPCB	Electronic

## 5 Build instructions

As most electronic components in the proposed module can be assembled by the manufacturer during PCB customization, this instruction provides key considerations if manual solder is needed due to manufacturer lacks sufficient stock of certain components.

• All resistors and compositors on the PCB are in 0805 size for easier hand-solder. When choosing resistors, please note that the output accuracy of Howland current sources is significantly affected by the resistor tolerance. Therefore, when purchasing, prioritize low tolerance (<1%) surface mount resistors (SMDs).
• If manual soldering is needed, carefully check the resistor positions and resistance values in the BOM and PCB layout to avoid mismatch, related files can be found here: https://github.com/DigitalNatureGroup/Howland-VCCS-for-GVS-experiment-prototype/tree/main/schematics or Zenodo: https://doi.org/10.5281/zenodo.14545604.

## 6 Operation instructions

After connecting the GVS module with additional components depicted in Fig. 5, follow the preparation steps below to ensure the system operates correctly. A multimeter is required to perform safety tests before involving human participants. For a more detailed analysis of the system’s performance, an oscilloscope is highly recommended. The calibration process also requires a few resistors (up to 5 kΩ) to simulate tissue impedance.

**Fig. 6:**
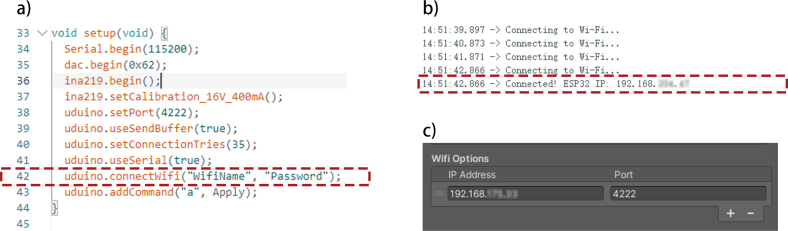
Arduino and Unity interface setup for GVS module communication. (a) Configuration of Wi-Fi credentials in Arduino setup code; ensure both the GVS module and PC are connected to the same network. (b) Arduino serial monitor displaying successful Wi-Fi connection and IP address. (c) Unity interface Wi-Fi settings configured to match the Arduino’s IP address, the default port is 4222.

**Fig. 7:**
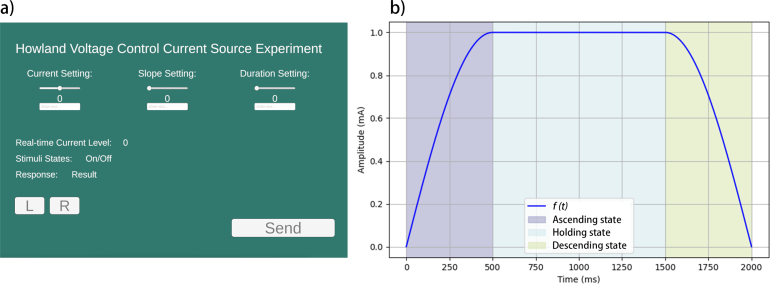
Interface and demonstration for different states of the stimulation: (a) PC-based graphical interface for wireless control of the GVS module, and (b) visualization of the stimulation current amplitude. The f(t) depicting ascending, holding, and descending states over time.

• This instruction is intended for the main demonstration, which is a standard tDCS application. The stimulation is controlled through a customized PC interface developed in Unity, allowing users to adjust the current amplitude, slope, and duration, as shown in Fig. 7. For other functions, please refer to the examples available in the corresponding GitHub repository.
• Upload the Arduino script (“Script.ino”) to MCU, communication between Arduino and Unity is achieved using the Uduino plugin, which can be accessed from this link [40].
• In the Script.ino file, ensure that the line uduino.connectWifi(“UserName”, “Password”); is updated with the correct credentials for the designated WiFi network (Fig. 6.a). Ensure that both the PC hosting the interface and the device are connected to the same WiFi network to enable data communication.
• After completing the above steps, the device’s IP address will appear on the Arduino serial monitor (Fig. 6.b). Open the Unity interface and enter the displayed IP address into the Uduino plugin. Restart the device and activate Unity’s Play Mode. The connection will be confirmed when the device, identified as Uduino_Wifi (defined in the code as uduino(“DeviceName”);), is successfully recognized (Fig. 6.c).
• For offset calibration, connect one resistor (with measured resistance similar to tissue impedance between mastoids, i.e. around 0.8 kΩ to 3 kΩ) and a multimeter (preferably in the microampere range) to the two electrode output terminals of the GVS module. Measure the error when the device is powered on (the default value should be 0 mA, error here refers to the difference between the actual measured value and default value). In the Arduino script, update the value “float offset” to the measured error. This step serves as a calibration procedure and can be repeated until the measured value approaches 0 mA.
• After completing the calibration, use the Unity interface to input various current values as desired, and observe the actual output on the multimeter. Verify whether the measured current aligns with the pre-set values within the acceptable experimental tolerance. If so, the output terminals can be connected to the electrode interface and may proceed to human trials.
• Before attaching the electrodes to the skin, it is recommended to use skin preparation gels to pre-treat the electrode placement area. This helps reduce the impedance of the skin and minimizes the sensation of tingling caused by electrical stimulation.
• For each test that involving participants, it is recommended to set the current intensity from ±0.74 mA (see the conclusions of the “User Test” section) to avoid discomfort caused by excessive current stimulation. If the participant reports no perception of the tilting sensation, the current intensity can gradually increase, with increments of 0 mA per adjustment.

## 7 Validation and characterization

### 7.1 Hardware performance test

We conducted two tests regarding the stability and settling speed of the current output. The results presented in this section not only demonstrated the actual performance of this module but also showed potential fluctuations and deviations in the output, which can be considered as potential variability and serve as sources of bias in future experiments.

#### 7.1.1 Precision and stability test (P&ST)

**Fig. 8:**
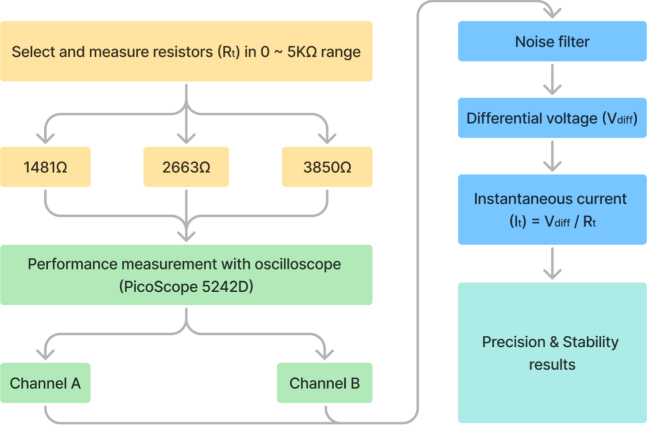
Diagram illustrating the P&ST test procedure.

When using the GVS module to generate a continuous current with fixed amplitude over a certain stimulation period, precision and stability are important. Assessing these parameters is critical for ensuring both the safety of the stimulation and the reproducibility of the experimental results. The proposed GVS module delivers current by leveraging the voltage differential between two electrodes. To evaluate system performance, the voltage differential between the electrodes was measured using a PicoScope 5242D oscilloscope. The corresponding current output was then calculated from these measurements by applying Ohm’s law. To simulate tissue impedance between mastoids, we used three resistors with measured resistances of 1481 Ω, 2663 Ω, and 3850 Ω, connected between the module electrodes. The voltage output from both electrodes was recorded under each of these resistance conditions to assess the performance of the proposed module across varying load impedance levels (Fig. 8).

The control signal was generated using a micro controller unit (MCU) with custom Arduino scripts (see attachment). For each load condition, the module was evaluated in current settings ranging of ±3.5 mA, with increments of 0.5 mA. The EHCS is highly sensitive to resistor tolerance, a offset error may exist. Before evaluation, we measured our module with a Sanwa CD771 digital multimeter and identified an offset error of 0.04 mA. To eliminate this, a manual offset adjustment was applied through the Arduino script. Notice that this offset error may vary between modules due to differences in manufacturing and deviation in resistance, therefore, it is essential to test each module before use to ensure optimal accuracy.

A PC-based user interface was designed using Unity (Version 3.5) to control current output through Wi-Fi (Fig. 7a). This interface was initially made for a fixed-current stimulation experiment, the same as the one used in the user test. It allows real-time control over the current’s amplitude, slope, and duration. Here, amplitude (A) refers to the desired current intensity. The slope (S) is the range of the ascending and descending states, aimed at preventing sudden stimulation, which causes tingling and burning sensations. Adding these transition states allows users to focus only on tilting perception. Duration (D) represents the length of the holding state, equal to the duration after the current reaches the desired intensity. Using the above parameters, a raised sine-like function was employed to generate the stimulation waveform shown in Fig. 7b. When using t (ms) as the time step, the waveform generation function can be expressed using the following equation, ranging from 0 to 2S+D.

(9) $$f\left(t\right)=\left\{\begin{array}{cc} A\cdot \sin\left(\frac{\pi }{2}\cdot \frac{t}{S}\right)\; & \text{if}0\leq t<S\;\text{(Ascending state)}\\ A\; & \text{if}S\leq t<S+D\;\text{(Holding state)}\\ A\cdot \sin\left(\frac{\pi }{2}-\frac{\pi }{2}\cdot \frac{t-\left(S+D\right)}{S}\right)\; & \text{if}S+D\leq t<2S+D\;\text{(Descending state)} \end{array}\right)$$

Fig. 8 illustrates the step-by-step procedure of the precision and stability (P&ST) test. Time-series voltage data from the electrodes were recorded using a dual channel setup with a sampling rate of 50 kS/s. Since the objective is to evaluate the accuracy and stability after the current reaches a certain level, the following tests focus only on the holding state.

**Fig. 9:**
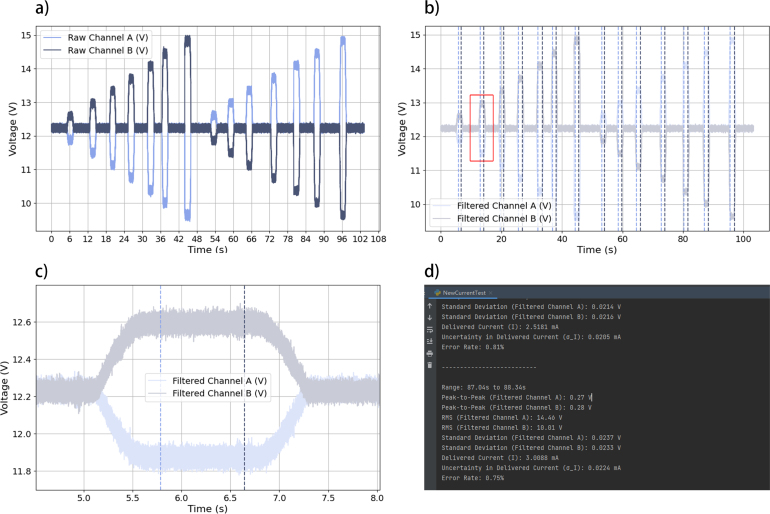
Evaluation procedure for the precision and stability test: (a) Raw data recorded from the oscilloscope, (b) filtered data with annotated segment markers for analysis, (c) segmentation of marked data intervals for further statistical evaluation, and (d) analysis results displayed on the Python console.

We identified inherent noise by recording signals while the GVS module was in non-activated state, raw data were recorded without applying any bandwidth limit and low-path filter from the oscilloscope (Fig. 9a). Instated, we use the Butterworth low-pass filter to process the raw data using Python (Version 3.9) to reduce the noise without sacrificing the integrity of the original signal.

The filter cutoff frequency was set to 10 kHz, with a filter order of 5. The fifth-order Butterworth filter’s frequency response is characterized by Eq. (10).

(10) $$\left|H\left(e^{j\omega_{d}}\right)\right|=\frac{1}{\sqrt{1+{\left(\frac{\omega_{d}}{\omega_{c}}\right)}^{2N}}},\;\omega_{c}=\frac{2f_{c}}{f_{s}}\;\text{where}N=5,\;f_{c}=10\text{kHz},\;f_{s}=50\text{kHz}$$

The filtering process was applied using a zero phase forward and reverse digital filtering technique (filtfilt), this ensures that no phase distortion occurs in the filtered signals (Fig. 9b). After filtering the raw data, a voltage threshold was set to distinguish the data segments of the holding state from other states for data analysis (Fig. 9c).

To evaluate the stability and reliability of the GVS module when delivering current to tissue, several key metrics were calculated from the filtered output voltage signals, including peak to peak (peak-peak), root mean square (RMS), and standard deviation (SD) values. Furthermore, the uncertainty in the current ($\sigma_{I}$) was derived based on the SD of the voltage signals and the known resistance (tissue simulation). The error rate provides an indication of the reliability of the measurement, was also calculated by comparing the uncertainty with the actual current (Fig. 9d).

**Fig. 10:**
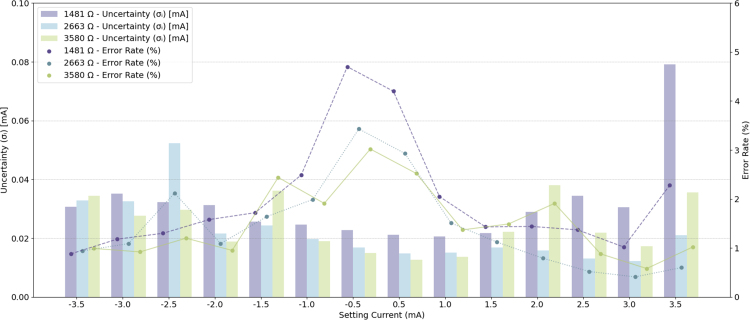
Precision and stability performance under various current and resistance settings. The uncertainty (σ, in mA) and error rate (in %) are shown across different current levels and load resistances (1481 Ω, 2663 Ω, and 3580 Ω). The error rate is plotted on the secondary axis.

Fig. 10 presents the performance of the proposed GVS module in terms of precision and stability in varying current and resistance settings. The delivered current was consistent across the setting range of ±3.5 mA. For each test duration, the SD of both electrodes’ output was observed to be higher in high-current settings (2.5 mA to 3.5 mA range, SD: 0.032 V to 0.094 V) compared to low-current settings (±0.5 mA to ±1.5 mA range, SD: 0.022 V to 0.032 V), suggesting that voltage fluctuations increase with higher current levels, potentially attributable to factors such as increased power dissipation and noise, which are less pronounced at lower current levels. The uncertainty ($\sigma_{I}$) of the output current ranged between 0.012 mA and 0.079 mA, with an error rate that ranged between 0.41% and 4.70% at the tested current levels. When comparing SD in different load conditions, higher loads (3850 Ω) were associated with greater waveform variations (SD: 0.040 V to 0.098 V) compared to lower loads (1481 Ω, SD: 0.022 V to 0.037 V). This indicates that higher load conditions result in increased waveform variability, likely due to greater impedance effects and heightened system noise. Across the three resistance values, the proposed GVS module demonstrated reliable performance, as it maintained a consistent current output compared to the current setting value. The results suggest robust control over the current delivery range under varying load conditions.

#### 7.1.2 Frequency response analysis

In some cases, stimulation, such as noisy galvanic vestibular stimulation (nGVS), often involves rapid changes in current amplitude with a zero mean characteristic [41]. This requires that the device possess the following abilities: short settling time, precise execution of input signals, and the ability to preserve the integrity of the desired waveforms. To evaluate these capabilities, a frequency response analysis (FRA) was performed to evaluate the performance of the proposed module in a frequency range of up to 640 Hz with the maximum amplitude of 1 mA, as this range is commonly used in studies related to nGVS [42].

To generate accurate zero-mean noise up to 640 Hz, compliance with the Nyquist criterion required a sampling rate of at least 1280 data points per second. Therefore, in this evaluation, we use the same 12-bit Adafruit MCP4725 DAC module to produce sine waves with 128 steps to ensure a smooth transition. Frequency sweeps were generated using a custom Arduino script (FunctionGeneratorExample.ino) to produce 20 linearly spaced frequency values, each repeating 3 times. The sine wave generation adhered to the Nyquist criterion, achieving 2560 samples per second at the maximum frequency of 20 Hz, exceeding the required minimum of 1280 samples per second.

Similarly to the previous P&ST, the FRA was also carried out under conditions designed to replicate the varying tissue impedance, using resistors with measured values of 1481 Ω, 2663 Ω, and 3850 Ω. The oscilloscope data was merged and the analysis was limited to the segment corresponding to the 1–20 Hz frequency sweep. The median offsets for both channels were calculated using data from an initial time range and subtracted to eliminate DC bias. To mitigate noise that could interfere with the FRA, voltage signals were down-sampled by a factor of 10 using a finite impulse response (FIR) filter-based decimation technique.

**Fig. 11:**
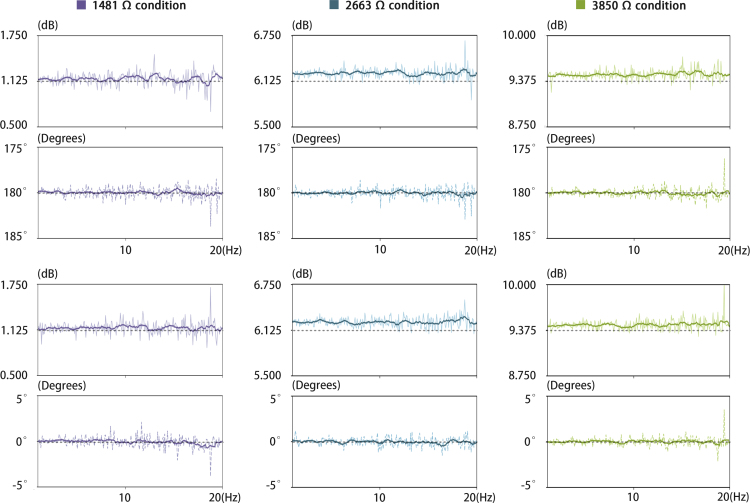
Bode plot of FRA in 1 Hz to 20 Hz range.

To quantify the system response, the decimated signals were analyzed using a fast Fourier transform (FFT). The gain in dB and the phase difference between the DAC (input signal) and the two electrodes (output signal) were calculated for frequencies between 1 Hz and 20 Hz. For easier observation of the trend, a moving average filter was applied to smooth the gain and phase difference spectra, the window size for smoothing was based on the frequency resolution. To evaluate performance, the gains of each scenario were compared with the LTspice simulation results using the following equation:

In Eq. (11), 12.3 V and 2.6 V represent the offsets of the electrode output and DAC output, therefore it is necessary to adjust the baseline to zero before performing the gain calculation. In which, simulation results shows that while with load resistance of 1481 Ω, 2663 Ω, and 3850 Ω, the expected gains are 1.152 dB, 6.251 dB, and 9.455 dB, respectively.

(11) $$G=20\cdot \log_{10}\left(\frac{V_{Output}-12.3}{V_{DAC}-2.6}\right)$$

The results demonstrated stable output performance for sine wave generation across the frequency range of 1 Hz to 20 Hz, with a maximum of 2048 samples per second. The magnitude responses were compared with the simulation results, revealing no significant attenuation throughout the range. Furthermore, phase differences between the two channels were observed to align with the expected values of 0°and 180°, as the system employs voltage differentials to generate current, resulting in the anticipated phase relationship for Channel A and Channel B. Furthermore, no significant delay was observed between different load conditions, indicating robust phase stability under varying system loads (Fig. 11).

### 7.2 User test

The objective of the user test is to evaluate the effectiveness of the hardware and identify the minimum distinguishable threshold of tilting sensation using the proposed waveform algorithm, thus establishing a reference setting for future users. The Just Noticeable Difference (JND) metric is a well-established method for quantifying perception thresholds. Since GVS lacks a visual indicator of functionality, JND provides a practical means to verify the performance of the prototype and facilitates replication by allowing comparison with existing data.

Thirteen participants (7 male, 6 female), aged 22 to 29 years (AVG = 25.75, SD = 2.49) from the University of Tsukuba were recruited for this experiment. A psychophysical approach was used to determine the JND. Specifically, a three-down, one-up (3D1U) staircase algorithm was used, with an initial stimulus of 1.6 mA and a step size of 0.1 mA. The minimal step size is determined by the uncertainty ($\sigma_{I}$) of current delivery from P&ST, which the maximum is 0.079 mA. The thresholding test employed a two-alternative forced-choice (2AFC) method, as the polarity of the current was found to influence participants’ perception of tilt direction.

**Fig. 12:**
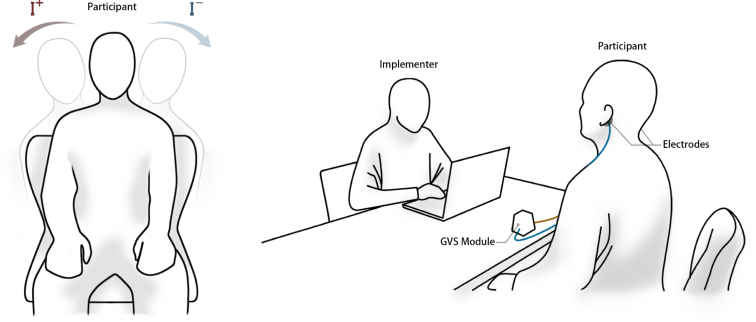
Experiment setting.

During the experiment, positive and negative stimuli were randomly presented. The polarity affects the direction of the tilting sensation (+ for tilting right, - for tilting left). When stimulation is provided, participants will perceive an illusion of tilting, allowing them to experience a shift in spatial orientation. Participants were required to indicate ‘left’ or ‘right’ based on their perceived direction of tilt (Fig. 12). The implementer assessed the correctness of each response and recorded the results, then repeat the above process. Participants remained unaware of the correctness of their response until the end of the experiment. The correctness of the response was also used to adjust the stimulation intensity for the subsequent trial: after three consecutive correct responses, the stimulation intensity was set to a lower step, while an incorrect response resulted in an increased step. The experiment was concluded after six reversals.

We conducted this test using the same waveform generation software as the P&ST, the slope was set at 500 ms, the duration was set at 1000 ms, resulting in a total stimulation duration of 2000 ms per trial. Fig. 6 shows the stimulation waveform while A = 1 mA, S = 1 ms and D = 1000 ms.

**Fig. 13:**
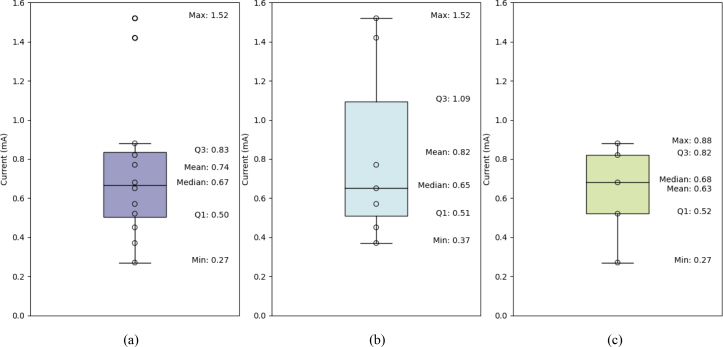
Minimal perceptual current level of GVS stimulation: (a) overall group, (b) male participants, and (c) female participants.

For all participants, Nuprep skin preparation gel (Weaver and Company, Houston, USA) was applied to the designated electrode sites to exfoliate the skin. After exfoliation, the gel and any residual material were thoroughly removed. The electrodes (MSGST-37 ECG electrodes) were then affixed bilaterally to the mastoid regions. Participants were instructed to conduct the test in a seated position, with their arms naturally resting on their thighs without supporting it, and also not to intentionally close their eyes during the test (Fig. 12).

During the experiment, one participant was unable to distinguish the direction of the tilt sensation. A total of 368 valid trials were conducted, with an average of 30.67 trials per participant (SD = 6.44). The minimum stimulus applied was 0.2 mA, while the maximum was 1.8 mA. The results for the remaining participants (7 male, 5 female) are presented in Fig. 13. The average JND is 0.74 mA with a standard deviation of 0.38 mA. In specific, the average JND for male participants (Mean: 0.82 mA; SD: 0.46 mA) is higher than for female participants (Mean: 0.63 mA; SD: 0.25 mA). However, no significant differences were found using the two-sample t-test (t (9.47) = 0.82, p = 0.43>0.05).

### 7.3 Conclusion

This study presents the design, implementation and evaluation of an open-source EHCS-based GVS module, developed to deliver precise, stable, and rapid-response electrical stimulation. In addition, the voltage-controlled approach enables researchers and content creators to customize stimulation parameters with minimal effort. The use of common development platforms further simplifies waveform definition, frequency adjustments, and current control. Our evaluation shows that this module demonstrates excellent performance under varying load conditions while maintaining compactness. This enables it to be integrated into other lightweight portable devices, such as XR head-mounted displays. This also promotes the conduct of tDCS-related experiments in non-lab environments.

To further enhance the module’s performance, addressing noise originating from the boost circuit and mitigating the effects of high load impedance is crucial, as these factors may adversely influence user experience. Future research should focus on how noise ripple specifically influences users’ subjective experiences, seek ways to improve user comfort in interaction and develop standardized benchmarks to evaluate hardware performance consistently.

Hardware performance tests demonstrated accurate current delivery, with the uncertainty of target current remaining minimal (Avg = 0.026, SD = 0.012). The observed levels of uncertainty in current delivery are probably attributed to noise generated by the boost circuits, the high load impedance used during testing, and external environmental factors such as electromagnetic interference. These results indicate that, while the system achieves high precision in current delivery, addressing noise and impedance effects could further enhance its performance in sensitive applications. The FRA demonstrates the module’s capability to handle rapid changes in current. The magnitude differences align closely with the simulation results in all load conditions, while the phase differences perform as expected (0°and 180°compared to the input signal), with no significant lag, ensuring reliable and precise current delivery under dynamic conditions. Results from hardware performance tests also show that the performance of the proposed module is highly dependent on the input signal (in this case, the DAC module), including output granularity, accuracy, and frequency. In addition, reserving an operational margin in the output range aids in calibration processes, but it also reduces the resolution of the delivered current.

Regarding the user test, the results demonstrate the effectiveness of the proposed hardware and waveform algorithm in eliciting tilting sensations through GVS. Using a psychophysical approach and a 3D1U staircase algorithm, the JND of tilt perception was determined. The use of a 2AFC paradigm and controlled stimulation parameters ensured consistent and reliable assessment of participants’ perceptions. The findings suggest an average JND of 0.74 mA (SD = 0.38), with male participants exhibiting a slightly higher threshold (Mean: 0.82 mA; SD: 0.46) compared to females (Mean: 0.63 mA; SD: 0.25). However, this difference was not statistically significant, as indicated by the independent t-test. These results provide a reference setting for future applications and experiments involving the proposed module, particularly in setting the initial value of the stimulation. Because minor limitations in noise and input signal were observed, these insights paved the way for optimizing the system for more sensitive and dynamic applications.

We hope that this study can streamline the prototyping cycle for GVS-related research and content development, providing researchers and developers with a reliable open-source platform to accelerate innovation and exploration in the field of GVS. By simplifying hardware integration and customization, this work aims to lower the entry barrier for designing and testing new GVS-based applications, fostering advances in both scientific understanding and practical implementations.

## CRediT authorship contribution statement

**Zhi Liu:** Writing – original draft, Visualization, Validation, Hardware design, Software, Methodology, Investigation, Data curation, Conceptualization. **Shieru Suzuki:** Writing – review & editing, Validation, Methodology, Investigation, Conceptualization. **Tatsuki Fushimi:** Writing – review & editing, Supervision, Methodology, Conceptualization. **Yoichi Ochiai:** Writing – review & editing, Supervision, Resources, Project administration, Funding acquisition, Conceptualization.

## Ethics statements

This study was conducted in accordance with the ethical guidelines established by the University of Tsukuba, Department of Informatics. Informed consent was obtained from all participants before the study began. Participants were informed of their right to withdraw from the study at any time without consequences.

## Declaration of competing interest

The authors declare the following financial interests/personal relationships which may be considered as potential competing interests: Zhi Liu reports financial support was provided by Pixie Dust Technologies, Inc. If there are other authors, they declare that they have no known competing financial interests or personal relationships that could have appeared to influence the work reported in this paper.
